# Clinical factors associated with in-hospital mortality in elderly versus non-elderly pneumonia patients in the emergency department

**DOI:** 10.1186/s12890-023-02632-z

**Published:** 2023-09-07

**Authors:** Brandon Chongthanadon, Netiporn Thirawattanasoot, Onlak Ruangsomboon

**Affiliations:** 1 Faculty of Medicine, Siriraj Hospital, Mahidol University, Bangkok, Thailand; 2 Department of Emergency Medicine, Faculty of Medicine, Siriraj Hospital, Mahidol University, 2 Wanglang Road, Bangkoknoi, Bangkok, 10700 Thailand

**Keywords:** Pneumonia, Elderly patients, Geriatric patients, Emergency department

## Abstract

**Background:**

Pneumonia is a respiratory infection with an increasing incidence with age. However, limited evidence has identified factors associated with its outcome among different age groups, especially in the elderly and in the emergency department (ED) setting. We aimed to identify clinical factors associated with in-hospital mortality in elderly versus non-elderly pneumonia patients in the ED.

**Methods:**

A retrospective observational study was conducted at the ED of Siriraj Hospital, Thailand. Patients aged at least 18 years old diagnosed with non-COVID pneumonia between June 1, 2021, and May 31, 2022, were included. They were categorized into the elderly (age ≥ 65 years) and non-elderly (age < 65 years) groups. The primary outcome was in-hospital mortality. We employed multivariate logistic regression models to identify independent factors associated with the outcome in each age group.

**Results:**

We enrolled 735 patients, 515 elderly and 222 non-elderly. There was no difference in in-hospital mortality rate between the two groups (39.0% in the elderly and 32.9% in the non-elderly; p = 0.116). In the elderly cohort, independent factors associated with in-hospital mortality were do-not-resuscitate (DNR) status (adjusted odds ratio (aOR) 12.89; 95% confidence interval (CI) 7.19–23.1; p < 0.001), Glasgow Coma Scale (GCS) score (aOR 0.91; 95%CI 0.85–0.96; p = 0.002), hemoglobin level (aOR 0.9; 95%CI 0.82–0.98; p = 0.012) and the type of initial oxygen support (p = 0.05). Among non-elderly patients, independent factors were DNR status (aOR 6.81; 95%CI 3.18–14.59; p < 0.001), GCS score (aOR 0.89; 95%CI 0.8–0.99; p = 0.025), platelet level (aOR 1; 95%CI 1–1; p = 0.038), Charlson Comorbidity Index (CCI) (aOR 1.12; 95%CI 0.99–1.28; p = 0.078), and the type of initial oxygen support p = 0.079).

**Conclusion:**

In pneumonia patients presenting to the ED, DNR status, lower GCS score, and more invasive initial oxygen supplementation were independently associated with in-hospital mortality in both elderly and non-elderly groups. However, lower hemoglobin level was only associated with in-hospital mortality in the elderly, while higher CCI and lower platelet count were independent factors only in the non-elderly. These findings emphasize the importance of age-specific considerations for the disease, and these factors are potential prognostic markers that may be used in clinical practice to improve patient outcomes.

## Introduction

Pneumonia is a respiratory infection that affects the lung tissue and can be caused by many types of organisms [1] In 2020, there were 195,729 pneumonia patients reported in Thailand, accounting for 294.89 cases per 100,000 populations [2]. In severe cases, patients may require invasive mechanical ventilation and potentially encounter unfavorable outcomes, especially mortality [3]. Pneumonia has also been one of the most common reasons for Emergency Department (ED) visits and remains an important cause of morbidity and mortality in adult patients [4]. With an increasing incidence with age, pneumonia portrays an even more critical health concern as the world population continues to age. Moreover, it tends to lead to more severe consequences in older patients. In Thailand, the mortality rate secondary to an infection caused by *S. pneumoniae* markedly increased with age, from 7.02% among patients 61–70 years old to 24.24% among those at least 81 years old [5]. Although the exact mechanisms underlying these findings are not fully understood, it is generally assumed that the aging process negatively impacts the lung function due to an increased work of breathing caused by reduced respiratory muscle strength, elastic recoil, and chest wall compliance [6]. These changes subsequently weaken the elderly’s ability to combat pulmonary infections effectively [6]. In addition, an increasing number of virulent organisms in older patients’ upper airways may further enhance their susceptibility to severe lower respiratory tract infections [7]. Given these circumstances, pneumonia will only become a more serious health issue as many countries undergo demographic shifts toward aging societies.

Several clinical factors have proven to be prognostic of adverse clinical outcomes secondary to pneumonia in adult patients, such as fever, male gender, hypoxemia, and various comorbidities [8–10]. Some laboratory values were also identified as potential prognostic markers, such as leukocytosis and elevated lactate dehydrogenase [8–10]. A study focusing on pneumonia patients of different age groups found that factors prognosticating mortality in younger pneumonia patients were malignancy and tachycardia, while male gender, congestive heart failure, blood urea nitrogen, hypoxemia, arterial pH, and pleural effusion were identified as prognostic factors in older patients [11]. In another study of Coronavirus 2019 (COVID-19) pneumonia patients in the ED, independent factors associated with in-hospital mortality in the elderly were age, male gender, do-not-resuscitate (DNR) status, body temperature, diastolic blood pressure, Glasgow Coma Score (GCS), C-reactive protein, and total bilirubin; in contrast, in the non-elderly, Charlson comorbidity index (CCI), oxygen saturation, procalcitonin, total bilirubin, and alanine aminotransferase were among potential prognostic factors [12]. The findings from these previous studies suggest that potential prognostic factors for pneumonia may vary across different age groups. However, there has been limited evidence on these factors focusing on elderly versus non-elderly pneumonia patients, especially in the ED setting. Therefore, we aimed to identify independent factors associated with in-hospital mortality in non-COVID pneumonia patients in the ED, stratified by elderly status. Specifically, we sought to compare and contrast these independent factors among elderly and non-elderly patients, as we hypothesized that different physiology between these age groups may result in different factors associated with poor clinical outcomes of pneumonia, especially mortality.

## Methods

### Study design and setting

This retrospective cohort study was conducted at the ED of Siriraj Hospital, the largest university hospital in Bangkok, Thailand, with over 20,000 triage level 1–2 ED visits per year. The study was performed in accordance with the Good Clinical Practice (GCP) guideline and the Declaration of Helsinki, and reported according to the STROBE checklist [13]. The study protocol was approved by Siriraj Institutional Review Board (certificate of approval: Si557/2022). Due to the retrospective nature of the study, patients’ informed consent was waived.

### Patients

Patients who visited the ED of Siriraj Hospital between June 1, 2021, and May 31, 2022, were screened for eligibility. Included in the study were those 18 years of age or older with clinical symptoms suggestive of pneumonia (namely fever, cough, and/or dyspnea) and a final diagnosis of pneumonia according to the ICD-10 codes upon discharge. Those with COVID-19 pneumonia or other conditions that were not pneumonia were excluded. After the patients’ inclusion, we classified them into the elderly group (those at least 65 years of age) and the non-elderly group (those less than 65 years old).

### Data collection and variables

We reviewed our ED patient registry and consecutively screened patients visiting the ED during the study period using their visiting symptoms and ICD-10 codes at discharge to identify eligible patients. After the patients’ inclusion, we reviewed their medical records and extracted the following variables: age, gender, underlying conditions, initial vital signs and mental status, initial laboratory results, type of pneumonia, type of oxygen support received at the ED and during hospital admission, ED disposition, ED and hospital length of stay, and in-hospital mortality. Initial respiratory rate was measured at triage before oxygen supplementation if not already received prior to hospital arrival. Furthermore, we calculated the severity of pneumonia based on the CURB-65 score [14]. All variables were electronically abstracted and recorded by a sixth-year medical student (BC), who was trained by the attending emergency physician researchers (NT and OR). One attending researcher (NT) later reviewed all recorded study data to ensure data completeness and accuracy, according to the recommended methodological standards for medical record review [15].

### Study objectives and outcomes

The primary objective of this study was to identify independent factors associated with in-hospital mortality among elderly and non-elderly emergency pneumonia patients. The primary clinical outcome was in-hospital mortality.

### Study size

We determined the sample size for this study based on logistic regression analyses associating potential clinical factors and in-hospital mortality in the elderly population. Fifteen variables were specified *a priori* as potential factors to be included in the logistic regression models, namely age, gender, CCI, DNR status, body temperature, blood pressure, pulse rate, respiratory rate, GCS score, hemoglobin level, white blood cell count, platelet count, renal function, type of pneumonia (3 subcategories), and type of initial oxygen support (5 subcategories). To ensure the model’s validity and avoid overfitting, a minimum of 10 events per variable (degree of freedom) or 190 outcome events were required to attain sufficient statistical power. The preliminary data of pneumonia patients in the ED of Siriraj Hospital in June 2022 indicated an overall mortality rate of 35% and a mortality rate of 30% and 38% in the non-elderly and elderly groups, respectively. Therefore, approximately 500 elderly patients were necessary to achieve adequate statistical power for the regression analysis. Based on the same preliminary data, we estimated from the prevalence of pneumonia patients that a data collection of 1 calendar year would provide an adequate number of elderly patients for the present study; we thus enrolled all eligible patients between June 2021 and May 2022.

### Statistical analyses

Categorical variables are presented as frequencies and percentages. We employed the Chi-squared or Fisher’s exact test to compare between-group differences as appropriate. Continuous variables are presented as mean and standard deviation (SD) or median and interquartile range (IQR) as appropriate, and they were compared using the Student’s t-test or Mann-Whitney U test, respectively. We compared patients’ characteristics by elderly and mortality status. Missing data are reported in the footnotes of each characteristics table. The rate of missingness was less than 1% for all variables; therefore, no maneuvers were employed to handle them, and we performed complete-case analyses.

To analyze independent factors associated with in-hospital mortality in each patient group (elderly and non-elderly), we employed a two-step approach. In the first step, univariate logistic regression analyses were used to identify potential associating factors within each age group. Variables with a p-value of less than 0.1 were included in the corresponding multivariate regression models. In the second step, multivariate logistic regression analyses were performed using the backward stepwise method (probability for stepwise entry and removal = 0.05 and 0.10, respectively) to identify independent factors associated with in-hospital mortality. As aforementioned, we determined *a priori* 15 variables that we would assess for their associations with in-hospital mortality. We only included one variable related to blood pressure (mean arterial pressure) and oxygenation (initial oxygen support type) to avoid multicollinearity in the multivariate models. The results from the univariate and multivariate models were reported as odds ratio (OR) and adjusted odds ratios (aOR), respectively, with 95% confidence intervals (CI).

All analyses were performed using SPSS 18.0 (IBM Corp., Chicago, IL). Unless otherwise specified, a p-value < 0.05 was considered statistically significant.

## Results

### Patient characteristics

A total of 842 patients presented to the ED of Siriraj Hospital from June 1, 2021, to May 31, 2022, with clinical symptoms suggestive of pneumonia and a negative test for COVID-19. Among these, 735 were diagnosed with pneumonia and were thus included in the study. Of all included patients, 56.2% were male, and their mean age was 71.2 years. The overall in-hospital mortality rate was 37.1%, and 23.4% of all patients required mechanical ventilation. Of the total participants, 69.8% (513 patients) were classified as the elderly group and the other 30.2% (222 patients) as the non-elderly group. Baseline characteristics by elderly status are presented in Table 1. In general, there were significantly higher prevalence of comorbidities and abnormal laboratory results in the elderly compared to the non-elderly group, except for malignancy. The elderly group also had significantly lower diastolic blood pressure (p = 0.001), pulse rate (p < 0.001), and GCS score (p = 0.001) than the non-elderly group, although other vital signs were not significantly different. Furthermore, the elderly had significantly higher severity based on the CURB-65 score than the non-elderly (p < 0.001), though this was not unexpected as age ≥ 65 is one of the score components. Regardless, patients with higher CURB-65 scores did not have a higher risk of in-hospital mortality than those with lower scores (Tables 2 and 3). No between-group difference in in-hospital mortality rate was seen despite a higher rate in the elderly (39% versus 32.9%: p = 0.116). However, the mechanical ventilation rate was significantly lower in the elderly than in the non-elderly (21.2% versus 28.4%: p = 0.037). Regardless, the elderly group had higher median length of hospital stay than the non-elderly, and more required hospital admission or died within the ED. In contrast, more non-elderly patients (6.3%) were discharged directly from the ED and treated on an outpatient basis than elderly patients (2.9%).

**Table 1:** Patient characteristics by age group (elderly versus non-elderly)

Characteristics	All patients (n = 735)	Age ≤ 65 years (n = 222)	Age > 65 years (n = 513)	p-value
Age (years)	71.2 ± 15	52.9 ± 10.5	79.1 ± 8.2	< 0.001
Sex (male)	413 (56.2)	132 (59.5)	281 (54.8)	0.257
**Underlying condition**				
Prior acute coronary syndrome	101 (13.7)	12 (5.4)	89 (17.3)	< 0.001
Prior cerebrovascular accident	183 (24.9)	26 (11.7)	157 (30.6)	< 0.001
Chronic lung diseases	176 (23.9)	46 (20.7)	130 (25.3)	0.189
Diabetes mellitus	236 (32.1)	60 (27)	176 (34.3)	0.058
Moderate to severe chronic kidney disease	146 (19.9)	21 (9.5)	125 (24.4)	< 0.001
Do-not-resuscitate status	410 (55.8)	92 (41.4)	318 (62.0)	< 0.001
Malignancy	247 (33.6)	96 (43.2)	151 (9.8)	< 0.001
Charlson Comorbidity Index	5.9 ± 2.8	4.6 ± 2.9	6.4 ± 2.5	< 0.001
**Vital signs and mental status**				
Body temperature (°C)	36.9, 1.1	37, 1.1	36.9, 1	0.432
Mean arterial pressure (mmHg)	95.7 ± 23.7	97.6 ± 24.4	94.9 ± 23.4	0.15
Pulse rate (beats/min)	103.5 ± 24.7	110.2 ± 23.7	100.6 ± 24.6	< 0.001
Respiratory rate (breaths/min)	32, 8	33.7 ± 9.6	32.4 ± 8.2	0.072
Glasgow Coma Scale score	15, 5	15, 4	14, 5	0.001
Room air oxygen saturation	91, 12	91, 12	91, 11.8	0.607
**Laboratory results**				
Hemoglobin (g/dL)*	10.5 ± 2.6	10.9 ± 2.8	10.3 ± 2.5	0.004
White blood cells (×1000/µL)**	11.4, 8.1	11.2, 7.8	11.4, 8.3	0.728
Platelet (×10,000/µL)*	26.2 ± 14.4	28.5 ± 15.6	25.2 ± 13.7	0.006
Glomerular filtration rate (ml/min/1.73m^2^)***	70 ± 37.8	70 ± 37.8	70 ± 37.8	70 ± 37.8
**Type of pneumonia**				0.465
Community-acquired pneumonia	437 (59.5)	130 (58.6)	307 (59.8)	
Hospital-acquired pneumonia	290 (39.5)	88 (39.6)	202 (39.4)	
Ventilator-associated pneumonia	8 (1.1)	4 (1.8)	4 (0.8)	
Severity of Pneumonia (CURB-65)****				< 0.001
CURB-65 score 0–1	87 (12.0)	60 (27.5)	27 (5.3)	
CURB-65 score 2–3	473 (65.1)	150 (68.8)	323 (63.5)	
CURB-65 score 4–5	167 (23)	8 (3.7)	159 (31.2)	
**Initial oxygen support**				0.113
None	89 (12.1)	30 (13.5)	59 (11.5)	
Cannula	269 (36.6)	80 (36)	189 (36.8)	
Mask with bag	242 (32.9)	61 (27.5)	181 (35.3)	
Endotracheal tube	75 (10.2)	30 (13.5)	45 (8.8)	
**Emergency Department disposition**				0.005
Inpatient ward	509 (69.3)	143 (64.4)	366 (71.3)	
Death	107 (14.6)	27 (12.2)	80 (15.6)	
Discharge	29 (3.9)	14 (6.3)	15 (2.9)	
**Oxygen support during hospitalization**				
Non-invasive ventilation	26 (3.5)	12 (5.4)	14 (2.7)	0.083
High-flow nasal cannula	113 (15.4)	40 (18)	73 (14.2)	0.22
Mechanical ventilation	172 (23.4)	63 (28.4)	109 (21.2)	0.037
**Outcome**				
Emergency department length of stay (hours)	14, 21	13, 17.5	15, 21.5	0.157
Hospital length of stay (days)	5, 11	4, 10	5, 11	0.033
In-hospital mortality	273 (37.1)	73 (32.9)	200 (39.0)	0.116

Note: data presented as n (%), mean ± standard deviation or median, interquartile range. The ‘others’ category of initial oxygen support and Emergency Department disposition is not shown in the table.

* Data missing for 2 patients in non-elderly group and 3 patients in elderly group.

** Data missing for 2 patients in non-elderly group and 4 patients in elderly group.

*** Data missing for 3 patients in non-elderly group and 2 patients in elderly group.

****Data missing for 4 patients in non-elderly group and 4 patients in elderly group, all due to missing Blood Urea Nitrogen.

**Table 2:** Elderly patient characteristics by in-hospital mortality status

Characteristics	Alive (n = 313)	Dead (n = 200)	p-value
Age (years)	78.3 ± 7.8	80.3 ± 8.8	0.008
Sex (male)	175 (55.9)	106 (53)	0.526
**Underlying condition**			
Charlson comorbidity index	6.2 ± 2.5	6.8 ± 2.5	0.01
Do-not-resuscitate status	137 (43.8)	181 (90.5)	< 0.001
**Vital signs and mental status**			
Body temperature (°C)	37, 1.2	36.8, 0.9	0.002
Mean arterial pressure (mmHg)	97.4 ± 22.1	90.9 ± 25	0.002
Pulse rate (beats/min)	99.3 ± 24	102.7 ± 25.3	0.122
Respiratory rate (breaths/min)	31.9 ± 8	33.2 ± 8.5	0.085
Glasgow coma scale score	15, 4	12, 6	< 0.001
Room air oxygen saturation	93, 9.8	88, 13.8	< 0.001
**Laboratory results**			
Hemoglobin (g/dL)*	10.6 ± 2.5	9.9 ± 2.4	0.001
White blood cells (×1000/µL)**	11.1, 8.1	12, 8.4	0.348
Platelet (×10,000/µL)*	25.3 ± 13.7	25.1 ± 13.7	0.901
Glomerular filtration rate (ml/min/1.73m^2^)***	64.4 ± 32.5	59.8 ± 33.9	0.124
**Type of pneumonia**			0.063
Community-acquired pneumonia	200 (63.9)	107 (53.5)	
Hospital-acquired pneumonia	111 (35.5)	91 (45.5)	
Ventilator-associated pneumonia	2 (0.6)	2 (1)	
Severity of Pneumonia (CURB-65)****			0.427
CURB-65 score 0–1	19 (6.1)	8 (4.0)	
CURB-65 score 2–3	199 (64.2)	124 (62.3)	
CURB-65 score 4–5	92 (29.7)	67 (33.7)	
**Initial oxygen support**			0.005
None	38 (12.1)	21 (10.5)	
Cannula	126 (40.3)	63 (31.5)	
Mask with bag	92 (29.4)	89 (44.5)	
Endotracheal tube	27 (8.6)	18 (9)	
**Outcome**			
Emergency department length of stay (hours)	13, 19.5	19, 31.8	< 0.001
Hospital length of stay (days)	7, 13	3.5, 6.8	0.001

Note: data presented as n (%), mean ± standard deviation or median, interquartile range. The ‘others’ category of initial oxygen support is not shown in the table.

* Data missing for 2 patients in alive group and 1 patient in dead group.

** Data missing for 3 patients in alive group and 1 patient in dead group.

*** Data missing for 2 patients in alive group.

**** Data missing for 3 patients in alive group and 1 patient in dead group.

**Table 3:** Non-elderly patient characteristics by in-hospital mortality status

Characteristics	Alive (n = 149)	Dead (n = 73)	p-value
Age (years)	51.8 ± 11.2	55.3 ± 8.3	0.01
Sex (male)	87 (58.4)	45 (61.6)	0.665
**Underlying condition**			
Charlson comorbidity index	4.1 ± 2.9	5.7 ± 2.7	< 0.001
Do-not-resuscitate status	38 (25.5)	54 (74)	< 0.001
**Vital signs and mental status**			
Body temperature (°C)	37, 1.2	37, 1.1	0.454
Mean arterial pressure (mmHg)	100.5 ± 23.8	91.8 ± 24.6	0.013
Pulse rate (beats/min)	110.3 ± 22	110.1 ± 26.9	0.952
Respiratory rate (breaths/min)	34.2 ± 10.3	32.6 ± 7.8	0.247
Glasgow coma scale score	15, 0.5	14, 6	< 0.001
Room air oxygen saturation	93, 11.5	88.5, 13.5	0.005
**Laboratory results**			
Hemoglobin (g/dL)*	11.2 ± 2.6	10.5 ± 3.1	0.085
White blood cells (×1000/µL)	11, 7.6	11.5, 9	0.648
Platelet (×10,000/µL)*	30.6 ± 14.5	24.4 ± 17.1	0.005
Glomerular filtration rate (ml/min/1.73m^2^)**	88.8 ± 43.3	84.1 ± 40.2	0.443
**Type of pneumonia**			0.223
Community-acquired pneumonia	93 (62.4)	37 (50.7)	
Hospital-acquired pneumonia	54 (36.2)	34 (46.6)	
Ventilator-associated pneumonia	2 (1.3)	2 (2.7)	
Severity of Pneumonia (CURB-65)***			0.899
CURB-65 score 0–1	41 (27.7)	19 (27.1)	
CURB-65 score 2–3	101 (68.2)	49 (70)	
CURB-65 score 4–5	6 (4.1)	2 (2.9)	
**Initial oxygen support**			0.001
None	27 (18.1)	3 (4.1)	
Cannula	57 (38.3)	23 (31.5)	
Mask with bag	30 (20.1)	31 (42.5)	
Endotracheal tube	18 (12.1)	12 (16.4)	
**Outcome**			
Emergency department length of stay (hours)	12, 16	15, 22.5	0.146
Hospital length of stay (days)	5, 10.5	3, 7	0.757

Note: data presented as n (%), mean ± standard deviation or median, interquartile range. The ‘others’ category of initial oxygen support is not shown in the table.

* Data missing for 1 patient in alive group and 1 patient in dead group.

** Data missing for 1 patient un alive group and 2 patients in dead group.

*** Data missing for 1 patient un alive group and 4 patients in dead group.

### Factors associated with mortality

Characteristics of elderly and non-elderly patients by in-hospital mortality status are presented in Tables 2 and 3, respectively. We only compared the characteristics that were determined *a priori* as potential clinical factors associated with the primary clinical outcome. In both age groups, patients who died upon hospital discharge were significantly older within their respective age groups and had higher CCI and lower blood pressure, GCS score, and oxygen saturation. Also, more of them had DNR status and required more invasive oxygen support than those discharged alive.

Independent factors and all other variables included in the univariate and multivariate logistic regression models for elderly and non-elderly patients are presented in Tables 4 and 5, respectively. DNR status and GCS score were identified as independent factors associated with in-hospital mortality in both elderly and non-elderly patients. Also, more severe and invasive initial oxygen support type was independently associated with in-hospital mortality in both groups. In the elderly, apart from DNR status (aOR 12.89; 95%CI 7.19–23.1; p < 0.001), GCS score (aOR 0.91; 95%CI 0.85–0.96; p = 0.002), and initial oxygen support type (p = 0.05), hemoglobin level was also independently associated with in-hospital mortality (aOR 0.9; 95%CI 0.82–0.98; p = 0.012). Whereas in the non-elderly, CCI (aOR 1.12; 95% CI 0.99–1.28; p = 0.078) and platelet count (aOR 1.0; 95% CI 1.0–1.0; p = 0.038) were independent factors associated with in-hospital mortality other than DNR status (aOR 6.81; 95%CI 3.18–14.59; p < 0.001), GCS score (aOR 0.89; 95%CI 0.8–0.99; p = 0.025), and initial oxygen support type (p = 0.079).

**Table 4:** Clinical factors associated with in-hospital mortality in elderly pneumonia patients

Factors	Univariate analysis (OR, 95%CI)	p-value	Multivariate analysis (aOR, 95%CI)	p-value
Age (years)	1.03 (1.01–1.05)	0.006	n/a	n/a
Sex (male)	0.89 (0.62–1.27)	0.518	n/a	n/a
Charlson comorbidity index	1.1 (1.02–1.18)	0.011	n/a	n/a
Do-not-resuscitate status	12.24 (7.26–20.64)	< 0.001	12.89 (7.19–23.1)	< 0.001
Body temperature (°C)	0.95 (0.85–1.07)	0.389	n/a	n/a
Mean arterial pressure (mmHg)	0.99 (0.98-1)	0.002	n/a	n/a
Pulse rate (beats/min)	1.01 (1-1.01)	0.122	n/a	n/a
Respiratory rate (breaths/min)	1.02 (1-1.04)	0.088	n/a	n/a
Glasgow coma scale score	0.87 (0.82–0.92)	< 0.001	0.91 (0.85–0.96)	0.002
Hemoglobin (g/dL)	0.89 (0.83–0.96)	0.002	0.9 (0.82–0.98)	0.012
White blood cells (×1000/µL)	1 (1–1)	0.478	n/a	n/a
Platelet (×10,000/µL)	1 (1–1)	0.901	n/a	n/a
Glomerular filtration rate (ml/min/1.73m2)	1 (0.99-1)	0.124	n/a	n/a
Type of pneumonia		0.064		n/a
Community-acquired pneumonia	Reference	-	n/a	n/a
Hospital-acquired pneumonia	1.53 (1.07–2.2)	0.021	n/a	n/a
Ventilator-associated pneumonia	1.87 (0.26–13.46)	0.535	n/a	n/a
Initial oxygen support		0.006		0.05
None	Reference	-	Reference	-
Cannula	0.91 (0.49–1.67)	0.749	1.13 (0.55–2.32)	0.742
Mask with bag	1.75 (0.95–3.21)	0.071	1.14 (0.56–2.31)	0.714
Endotracheal tube	1.21 (0.54–2.68)	0.646	2.44 (0.91–6.52)	0.076
Others	0.54 (0.22–1.36)	0.191	0.42 (0.15–1.19)	0.104

Abbreviation: OR, odds ratio; aOR, adjusted odds ratio; CI, confidence interval

**Table 5:** Clinical factors associated with in-hospital mortality in non-elderly pneumonia patients

Factors	Univariate analysis (OR, 95%CI)	p-value	Multivariate analysis (aOR, 95%CI)	p-value
Age (years)	1.04 (1.01–1.07)	0.022	n/a	n/a
Sex (male)	1.15 (0.65–2.03)	0.643	n/a	n/a
Charlson comorbidity index	1.21 (1.1–1.34)	< 0.001	1.12 (0.99–1.28)	0.078
Do-not-resuscitate status	8.3 (4.38–15.74)	< 0.001	6.81 (3.18–14.59)	< 0.001
Body temperature (°C)	0.85 (0.63–1.15)	0.288	n/a	n/a
Mean arterial pressure (mmHg)	0.99 (0.97-1)	0.014	n/a	n/a
Pulse rate (beats/min)	1 (0.99–1.01)	0.948	n/a	n/a
Respiratory rate (breaths/min)	0.98 (0.95–1.01)	0.248	n/a	n/a
Glasgow coma scale score	0.85 (0.79–0.93)	< 0.001	0.89 (0.8–0.99)	0.025
Hemoglobin (g/dL)	0.91 (0.82–1.01)	0.087	n/a	n/a
White blood cells (×1000/µL)	1 (1–1)	0.929	n/a	n/a
Platelet (×10,000/µL)	1 (1–1)	0.006	1 (1–1)	0.038
Glomerular filtration rate (ml/min/1.73m2)	1 (0.99-1)	0.442	n/a	n/a
Type of pneumonia		0.227		n/a
Community-acquired pneumonia	Reference	-	n/a	n/a
Hospital-acquired pneumonia	1.58 (0.89–2.81)	0.117	n/a	n/a
Ventilator-associated pneumonia	2.51 (0.34–18.51)	0.366	n/a	n/a
Initial oxygen support		0.002		0.079
None	Reference	-	Reference	-
Cannula	3.63 (1-13.16)	0.05	3.16 (0.77–13.03)	0.112
Mask with bag	9.3 (2.55–33.92)	0.001	3.48 (0.83–14.67)	0.089
Endotracheal tube	6 (1.48–24.3)	0.012	5.97 (1.17–30.35)	0.031
Others	2.12 (0.42–10.65)	0.363	1.03 (0.17–6.35)	0.979

Abbreviation: OR, odds ratio; aOR, adjusted odds ratio; CI, confidence interval

## Discussion

The objective of our study was to comprehensively evaluate clinical factors associated with in-hospital mortality in pneumonia patients, specifically comparing those among the elderly and non-elderly populations. First, we found no significant difference in in-hospital mortality rate between the two populations, and age was not independently associated with in-hospital mortality in both groups, despite some previous studies demonstrating an association between increasing age and higher mortality rates in pneumonia patients [16, 17]. Regardless, our findings might have aligned with a study of pneumonia patients in Argentina, which found that age did not have an impact on mortality in patients with one comorbidity or less, except for those aged above 80 [18]. Similarly, a study involving pneumonia patients in Singapore suggested that age only increased mortality risk in patients above 85 years old [11]. Based on these previous findings, we should expect to see a positive association between age and mortality in the extreme elderly, who experience a drastic decline in immune response and lung function, consequently resulting in higher mortality rates with increasing age. Consequently, it should not be surprising that we did not find such an association in our elderly patients, with a mean age of only 79.1 years old. Nevertheless, there could still have been some interplay between age and comorbidities in association with mortality risk in pneumonia patients of each age group. However, we did not have enough sample to explore those potential correlations and associations in this study.

In the present study, we aimed to identify independent factors associated with in-hospital mortality among the two age populations for two main reasons; to better understand the different characteristics and physiology between the two age strata and to employ these factors as potential prognostic factors that can guide patient triage, treatment, and disposition plan in order to prevent such an adverse consequence. Since only limited evidence existed, especially in the ED setting, the results of the present study have added to the current body of evidence and answered these two important gaps of knowledge. We demonstrated that although many clinical factors independently and strongly associated with in-hospital mortality were the same in both groups, there were some distinct characteristics and potential predictors of the outcome among these two age groups.

In both the elderly and non-elderly patients, we observed a robust association between DNR status and in-hospital mortality, which were in line with previous studies in both non-COVID [19, 20] and COVID pneumonia patients [12], as well as in sepsis patients [21]. These concordant associations over a wide range of diseases are unsurprising as individuals who opt for DNR status often have detrimental underlying health conditions and poor prognoses. With impaired baseline status, they tend to develop the disease more easily and have worse clinical progression than healthier individuals without DNR status. Moreover, it is notable from the present study that more elderly patients had DNR status, corresponding to their lower mechanical ventilation rate, than the non-elderly, and the strength of association was also stronger in the elderly. Apart from DNR status, our study results also revealed a significant association between GCS score and in-hospital mortality, also in concordance with the previous studies [12, 21]. In fact, GCS score was the only initial vital variable that was significantly associated with the outcome in the present study, demonstrating its importance as a potential prognosticating factor that may help prioritize patients’ evaluation, treatment, and disposition in the ED. Moreover, we found that the type of initial oxygen support was independently associated with in-hospital mortality in both age groups, with more severe and invasive methods associated with higher odds of the outcome, which was as expected and not surprising considering the condition of interest was a pulmonary disease. Specifically, among the different forms of oxygen intervention, endotracheal intubation demonstrated the strongest association with in-hospital mortality, which could have been attributed to the fact that those requiring mechanical ventilation were likely to have more severe disease and a poorer prognosis. Consequently, the need for mechanical ventilation should undoubtedly serve as a key indicator of disease severity and a potential prognosticating factor of in-hospital mortality in emergency pneumonia patients.

Interestingly, while initial oxygen supplementation was found to be associated with in-hospital mortality, respiratory rate did not demonstrate a significant association. This discordance could have been because, in some patients, respiratory rate was measured after oxygen supplementation was already delivered from emergency medical services, thus probably not reflecting the true disease severity. It could have also been explained by the fact that desaturation seen in pneumonia patients is mainly caused by the disease itself, while respiratory rate may be influenced by multiple factors, such as pain, fever, and underlying comorbidities, all contributing to its variability [22]. Therefore, oxygen supplementation, a direct intervention aimed at improving oxygenation, involves more specific and targeted respiratory support and thus plays a crucial role in managing respiratory compromise secondary to pneumonia. On the other hand, respiratory rate alone may not always serve as a direct indicator of the severity of respiratory dysfunction in pneumonia patients. Consequently, initial oxygen management may exhibit a stronger association with in-hospital mortality compared to respiratory rate alone in pneumonia patients.

Apart from the three common factors, we observed that hemoglobin level was the only laboratory variable significantly associated with in-hospital mortality in the elderly group. This result was in agreement with a previous study that indicated anemia as a significant factor associated with mortality in patients of various conditions, including pneumonia [23]. Chronic diseases are known to trigger inflammatory processes that can lead to anemia by reducing the lifespan of red blood cells and impairing the response of red blood cell progenitors to erythropoietin [24]. Even after patients recover clinically from pneumonia, they could still have persistent subclinical inflammation [25]. Such chronic inflammation resulting from chronic underlying diseases, primarily found in the elderly population, could have explained the low hemoglobin level among elderly pneumonia patients and their vulnerability to a higher risk of mortality, thus explaining the established association in the present study.

On the other hand, CCI was the baseline variable significantly and independently associated with in-hospital mortality only in non-elderly pneumonia patients and not in the elderly, similar to a previous study [12]. CCI represents an inclusive reflection of patients’ underlying comorbidities and thus directly indicates the patients’ overall health status. Taking the previous study [12] and this study together, we may propose that CCI is an informative and valuable index for prognostication purposes in the non-elderly. However, such a circumstance may not sustain in the elderly population. Future studies are required to explore if other comprehensive underlying indices can have better prognostic utility in the elderly. Moreover, we found platelet count as the only laboratory variable independently associated with in-hospital mortality in non-elderly pneumonia patients. This finding aligned with a previous study reporting that abnormal platelet count, whether thrombocytopenia or thrombocytosis, was associated with mortality in pneumonia patients [26]. In our study, however, it can be seen that the association was between in-hospital mortality and low platelet level. It is recommended that clinicians investigate alternative explanations for thrombocytopenia when found in pneumonia patients, such as disseminated intravascular coagulation and severe sepsis [26]. These causes could potentially explain the occurrence of thrombocytopenia and its association with in-hospital mortality in the non-elderly subgroup within our study. On the contrary, age-related changes in platelet function, one of which is platelet hyperactivity in the elderly, could have explained the absence of such association in elderly patients in our study [27].

In addition, it is interesting to observe that the CURB-65 score, the most commonly-used severity score for pneumonia, was not associated with in-hospital mortality in both elderly and non-elderly patients. This finding was most likely due to the insignificant associations between most of the score’s components and the outcome in our population. This result also highlights the need for future studies to further evaluate the utility of risk scores of different components for pneumonia patients, especially in the elderly population.

### Limitations

This study had some limitations. First, it was a single-center study, which could limit the generalizability of our findings to other settings, as there were some characteristics specific to our setting. For example, we did not collect nor aim to report intensive care unit (ICU) admission rate because, due to limited ICU resources, most patients with pneumonia at our hospital are only admitted to internal medicine wards despite requiring mechanical ventilation or having mild to moderate acute respiratory distress syndrome or septic shock. Therefore, this variable would not have been informative nor generalizable. Second, we employed only one age cut-off and categorized patients into only two groups, partly because we had an inadequate sample size to do otherwise. Therefore, we might not have captured all relevant heterogeneity among different age strata, especially the extreme elderly patients. Also, the less-than-optimal sample size in the non-elderly group could have caused underpowered analyses, especially in the regression model. Third, with the study’s retrospective nature, we had limited available variables with complete and accurate data. We could have missed important outcomes of patients discharged from the ED, although we found most of these patients attending follow-up appointments without unexpected ED revisits within 72 h. Fourth, there could have been selection bias due to more non-elderly patients being treated on an out-patient basis. Future studies, preferably collecting data prospectively, incorporating multiple healthcare facilities with all relevant age strata should be conducted to address these limitations and fill the current knowledge gaps.

## Conclusion

The present study found no significant difference in in-hospital mortality rate among elderly and non-elderly emergency pneumonia patients. DNR status, lower GCS score, and more invasive initial oxygen supplementation were independently associated with in-hospital mortality in both elderly and non-elderly groups. However, lower hemoglobin level was only associated with in-hospital mortality in the elderly, while higher CCI and lower platelet count were independent factors only in the non-elderly. These findings emphasize the importance of age-specific considerations for the disease and in its management, and these factors serve as potential prognostic markers that may be used in clinical practice to improve patient outcomes.

## Data Availability

The datasets generated and analysed for this study are not publicly available but are available from the corresponding author upon reasonable request.

## Abbreviations

ED: emergency department
COVID-19: Coronavirus disease 2019
DNR: do-not-resuscitate
GCS: Glasgow coma Scale
CCI: Charlson comorbidity index
SD: standard deviation
IQR: interquartile range
OR: odds ratio
aOR: adjusted odds ratio
CI: confidence interval
ICU: intensive care unit
